# Protective effect of hot peppers against amyloid β peptide and brain injury in AlCl_3_-induced Alzheimer’s disease in rats 

**DOI:** 10.22038/IJBMS.2022.67871.14845

**Published:** 2023-03

**Authors:** Omar M.E. Abdel-Salam, Marwa El-Sayed El-Shamarka, Eman R Youness, Nermeen Shaffie

**Affiliations:** 1 Department of Toxicology and Narcotics, Medical Research, and Clinical Studies Institute, National Research Centre, Cairo, Egypt; 2 Department of Medical Biochemistry, Medical Research, and Clinical Studies Institute, National Research Centre, Cairo, Egypt; 3 Department of Pathology, Medical Research, and Clinical Studies Institute, National Research Centre, Cairo, Egypt

**Keywords:** Amyloid-Β peptide, Anti-oxidant, Capsaicin, Capsicum, Hot pepper, Memory, Neuroprotective

## Abstract

**Objective(s):**

This study investigated the therapeutic effect of red hot pepper (*Capsicum* annuum) methanolic extract in induced Alzheimer’s disease using AlCl_3_ in male rats.

**Materials and Methods:**

Rats were injected with AlCl_3_ intraperitoneally (IP) daily for two months. Starting from the 2nd month of AlCl_3_, rats received, in addition, IP treatments with *Capsicum *extract (25 and 50 mg/kg) or saline. Other groups received only saline or *Capsicum *extract at 50 mg/kg for two months. Brain levels of reduced glutathione (GSH), nitric oxide (NO), and malondialdehyde (MDA) were determined. Additionally, paraoxonase-1 (PON-1) activity, interleukin-6 (IL-6), Aβ-peptide, and acetylcholinesterase (AChE) concentrations in the brain were measured. Behavioral testing included wire-hanging tests for neuromuscular strength and memory tests such as Y-maze and Morris water maze. Histopathology of the brain was also done.

**Results:**

Compared with saline-treated rats, AlCl_3_ caused significant elevation of brain oxidative stress as GSH level and PON-1 activity were depleted along with MDA and NO level elevation in the brain. There were also significant increases in brain Aβ-peptide, IL-6, and AChE levels. Behavioral testing indicated that AlCl_3_ decreased neuromuscular strength and impaired memory performance. *Capsicum *extract given to AlCl_3_-treated rats significantly alleviated oxidative stress and decreased Aβ-peptide and IL-6 in the brain. It also improved grip strength and memory functioning and prevented neuronal degeneration in the cerebral cortex, hippocampus, and substantia nigra of AlCl_3_-treated rats.

**Conclusion:**

Short-term administration of ASA (50 mg/kg) has adverse effects on male reproductive function in mice. Co-administration of melatonin protects against ASA-induced impairment of male reproductive function by preventing the reduction in serum TAC and testosterone levels seen with ASA treatment alone.

## Introduction

Alzheimer’s disease (AD) is a progressive neurological disorder and the most common reason for severe memory impairment among the elderly worldwide (1). It is marked by the insidious onset of memory deficits which increase in severity over time and are associated with a progressive decline in cognitive functioning, eventually leading to profound dementia, complete incapacity, and death 7 to 10 years after diagnosis (2, 3). The neuropathologic hallmarks are amyloid β extracellular plaques and hyperphosphorylated microtubule-associated (tau) proteins that form intracellular neurofibrillary tangles (4). The basal forebrain cholinergic system, cortex, hippocampus, and amygdale are the most affected brain regions due to the accumulation of amyloid-β peptide (Aβ) deposits that arise from mutation in the amyloid precursor protein causing neuronal damage (4, 5). Impaired cholinergic neurotransmission in the forebrain and hippocampus due to deficits in the choline acetyltransferase enzyme and reduced acetylcholine (ACh) content is indicated (6). This is the basis for the use of cholinesterase inhibitors such as donepezil, galantamine, and rivastigmine, as the first-line treatment, which at best improves or stabilizes the deterioration in cognition, function, and behavior (7).

AD is a sporadic neurodegenerative disorder in the majority of cases (more than 95%), for which no cause can be found (8). In the search for a causative agent, aluminum (Al), the most abundant toxic metal on earth has been implicated as a possible etiological factor (9, 10). Animal research showed that Al causes memory deterioration, amyloid-beta deposits, and neurodegeneration (11, 12). The deposition of Aβ peptide and in particular the soluble Aβ oligomers are considered the most toxic species that initiate the process of neurodegeneration. The activation of microglia and astrocytes causes increased production of pro-inflammatory cytokines e.g., tumor necrosis factor-α (TNF-α) and interleukin-1β (IL-1β), superoxide radical, and nitric oxide. This results in oxidative stress and neuroinflammation and consequent neuronal cell death (13, 14). 

Since oxidative stress may have a significant role in the neurodegenerative process in AD, anti-oxidants could be one therapeutic approach to prevent disease progression (15). Hot red or green peppers of the plant genus *Capsicum *(*Capsicum annuum* and* Capsicum* *frutescens*) which belong to the *Solanaceae* family are the most consumed spices in the world (16). Hot peppers are used to add color and spicy flavor to food or to prepare exotic recipes at restaurants and at home (16, 17). Peppers are rich in carotenoids, anthocyanin flavonoids, phenolics, vitamin A, vitamin E (alpha-tocopherol), and vitamin C (ascorbate) and have been reported to have strong anti-oxidant activity (18). Chilli hot peppers are also the source of the alkaloid capsaicin or trans-8-methyl-*N*-vanillyl-6-noneamide which is the major active pungent agent (19). 

Studies have demonstrated neuroprotective effects for extracts of hot pepper in experimental models of Parkinson’s disease (20) and insulin-induced hypoglycemia (21). Moreover, the intake of a capsaicin-rich diet has been reported to result in better cognition and lower Aβ levels in the serum of people aged 40 years or more (22). Similar data were reported in the APP/PS1 genetic mouse model of AD (23). *Capsicum* thus may prove a beneficial nutraceutical to prevent and/or slow down neurodegeneration in the brain of patients with AD (22, 23). Therefore, in this study, we investigated a methanolic extract of red hot pepper as a potential therapeutic agent using the rat AlCl_3 _model of AD. 

## Materials and Methods

***Animals***

Adult male Sprague-Dawley rats, weighing about 160–180 g, were used in the present study. Animals were obtained from the animal facility of the National Research Center. After one week of accommodation, rats were group-housed under temperature and humidity-controlled conditions with 12 hr light/dark cycle and free access to standard laboratory food and water during the study. All instructions adhered to to ethical considerations in handling laboratory animals of the Institute Ethics Commitee of the of the National Research Centre and complied with the Guide for the Care and Use of Animals by the U.S. National Institutes of Health (Publication No. 85-23, revised 1996). 

***Drugs and chemicals***

Aluminum chloride, obtained from Sigma-Aldrich in the USA, was dissolved in saline and given to rats intraperitoneally (IP) daily at a dose of 10 mg/kg to induce AD. Other chemicals and reagents were supplied by Sigma Chemical Co, USA. *Capsicum* fruits (hot red peppers) were purchased from the local market in Egypt. *Capsicum* fruits were chopped into pieces, dried, and ground. 70% methanol was used in the extraction process. Reduced pressure and lyophilization were used in extract evaporation (20). Capsaicin in the pepper extract was previously quantified with high-performance liquid chromatography and found to be 1.2% (20, 21).

***Experimental design***

Rats were arranged randomly into control and four treated groups (eight rats per group). The Control group received 0.2 ml saline daily for 60 days. Group 2 was given saline daily for 30 days and then treated daily with *Capsicum* extract (50 mg/kg, IP) during the 2^nd^ month. The last three groups received daily IP injections of AlCl_3_ (10 mg/kg) for 60 days. Groups 4 and 5 were given *Capsicum *extract (25 or 50 mg/kg, IP) daily during the 2^nd^ month of the study. The behavioral tests were arranged from the least stressful to the most stressful test. Morris water maze (WMZ) was done in the last 5 days of treatments, while wire hanging and Y-maze tests were performed on the last 2 days of different treatments. At the end of the study, animals were anesthetized and then sacrificed using cervical dislocation. Brains were quickly removed, washed with ice-cold saline, and dried. One-half of the brain was weighed and stored at −80 °C for further biochemical studies. They were homogenized with 0.1 M phosphate-buffered saline (pH 7.4) for biochemical measurements. The other hemisphere was fixed in 10% formol/saline for histological staining (24).

***Biochemical assays***

*Lipid peroxidation*

Measuring the level of malondialdehyde (MDA) was used for lipid peroxidation determination according to Ruiz-Larrea *et al*. (25). Red colored TBA-MDA adduct was obtained due to the reaction of 2-thiobarbituric acid with MDA at 25 °C with a peak absorbance of 532 nm. 

*Nitric oxide*

Griess reagent was used for nitric oxide determination in the brain. In this assay, the enzyme nitrate reductase converted nitrate to nitrite. Then nitrite reacted with Griess reagent forming a purple azo compound, its absorption was measured at 540 nm (26). 

*Reduced glutathione*

Ellman´s reagent (DTNB; 5, 5’-dithiobis (2-nitrobenzoic acid)) reacts with the free thiol group of GSH forming 2-nitro-s-mercaptobenzoic acid producing yellow color with absorbance which is measured at 412 nm using spectrophotometer **(**27). 

*Amyloid Aβ peptide*

The determination of Aβ1-41 content in the brain tissue was carried out using rat amyloid beta peptide 1-41 (Aβ1-42) ELISA Kit (SinoGeneClon Biotech Co., Ltd, China) (28).

*Interleukin-6*

Estimation of IL-6 level in the brain was carried out using an ELISA kit (Glory Science Co, Ltd, Del Rio, TX, USA) according to the manufacturer’s instructions (29).

*Paraoxonase-1*

Phenylacetate as a substrate was used to determine the arylesterase activity of the paraoxonase-1 enzyme in brain tissue homogenates. Phenylacetate is hydrolyzed into phenol, hydrolysis rate was determined using a spectrophotometer by monitoring the absorbance increment at 270 nm. One unit of arylesterase activity was equivalent to 1 μM of phenol formed per minute. Enzyme activity expressed as kU/l was calculated based on the molar extinction coefficient of 1310 M^-1^ cm^-1^ for phenol, pH 8.0 and 25 °C (30).

*Acetylcholinesterase*

Acetylcholinesterase (AChE) level in brain tissue homogenate was determined using an ELISA kit supplied by NOVA (Bioneovan Co, Ltd, DaXing Industry Zone, China) (31).

***Behavioral testing***

*Wire hanging test*

To determine the effect of capsicum on motor strength, rats were allowed to hang by their forelimbs to a steel rod (25 cm long, 0.2 cm in diameter), 0.5 m above the bench. The time taken by rats to suspend from the rod was determined three times with a cut-off time of 180 sec (32).

*Water maze test*

Morris water maze test was used to determine the Spatial working memory (33). It consisted of a glass tank, that was 20 cm wide, 40 cm in height, and 70 cm in length, filled to a depth of 21 cm with water maintained at 25 °C. The escape platform was hidden from sight and placed 1 cm below the surface of the water. Time taken by rats to reach the hidden platform was recorded as an average for three consecutive trials. The cut-off time was 60 sec.

*Y-maze test*

Short-term memory (spatial memory) was estimated using the Y-maze test. It is a wooden maze having three arms: 40 cm long, 30 cm high, and 15 cm wide, extending from a central platform at 120 °. On the test day, each rat was placed in the middle of the maze and left to explore its arms for 8 min, the sequence of entries was determined. The rat with good memory is expected to explore all of the 3 arms equally without repeating. This is expressed in the form of spontaneous alternations percentage which was calculated using the formula [number of alternations/number of entries-2] X 100 (34).

*Histopathological assessment studies*

Brains of different groups were fixed in 10% formol saline for seventy-two hours. Washing was done in tap water then serial dilutions of alcohol (methyl, ethyl, and absolute ethyl) were used for dehydration. Specimens were cleared in xylene and embedded in paraffin. Serial sections 5 μm thick were cut and stained with hematoxylin and eosin (H&E). Sections were photographed and evaluated using a bright-field microscope (Optiphot 2; Nikon, Tokyo, Japan).

***Statistics***

Results were expressed as mean ± SE. Statistical significance was determined using one-way ANOVA, followed by Tukey’s multiple comparisons test for multiple group comparison using GraphPad Prism 6 for Windows (GraphPad Prism Software Inc, San Diego, CA, USA). A probability value of less than 0.05 was considered statistically significant.

**Figure 1:**
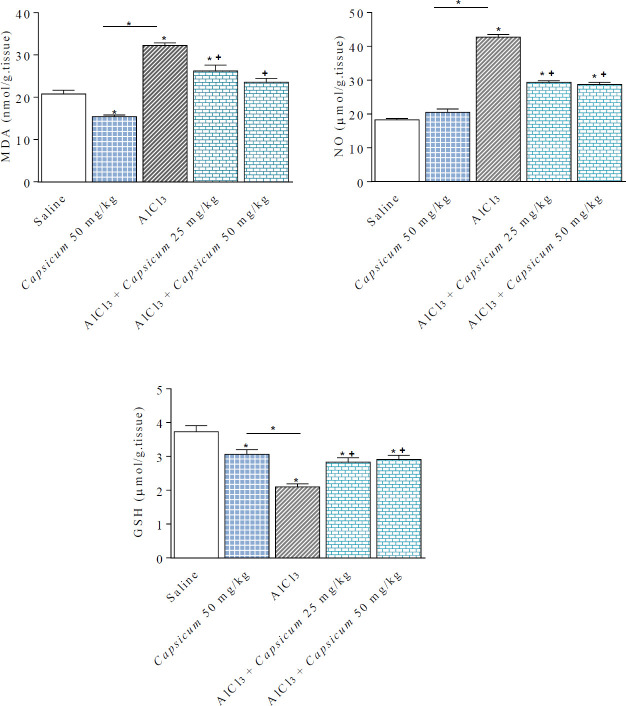
Effect of *Capsicum* on brain oxidative stress parameters in AlCl_3_-treated rats; malondialdehyde (MDA), nitric oxide (NO) and reduced glutathione (GSH). *: *P*<0.05 vs saline group and between different groups as shown in the graph. +: *P*<0.05 vs AlCl_3_ group

**Figure 2:**
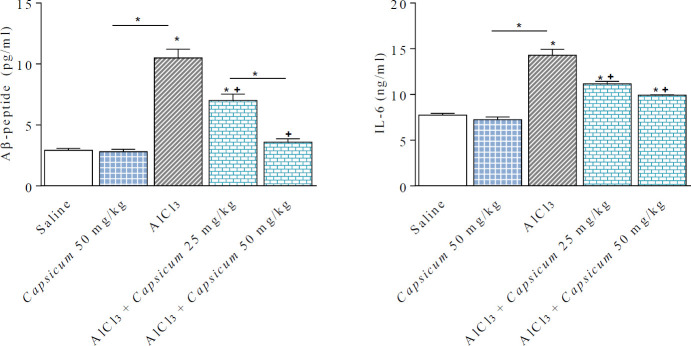
Effect of *Capsicum* on Aβ-peptide and IL-6 concentrations in brains of AlCl_3_-treated rats. *: *P*<0.05 vs saline control group and between different groups. +: *P*<0.05 vs AlCl_3_ group

**Figure 3:**
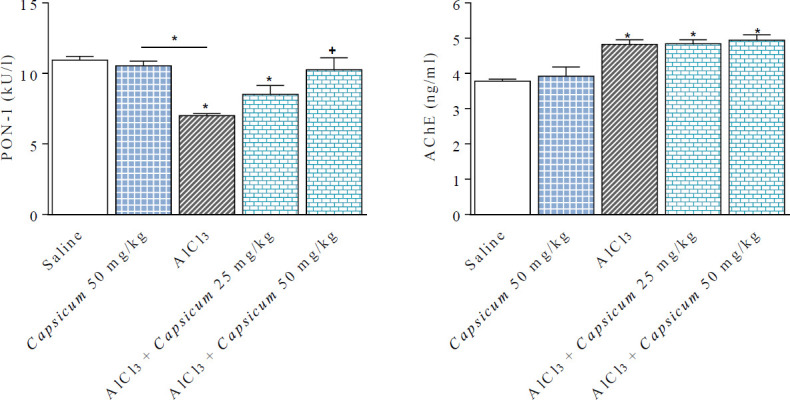
Effect of *Capsicum* on paraoxonase-1 (PON-1) activity and acetylcholinesterase (AChE) concentration in brains of different groups treated with AlCl_3_. *: *P*<0.05 vs saline group and between different groups. +: *P*<0.05 vs AlCl_3_ group

**Figure 4:**
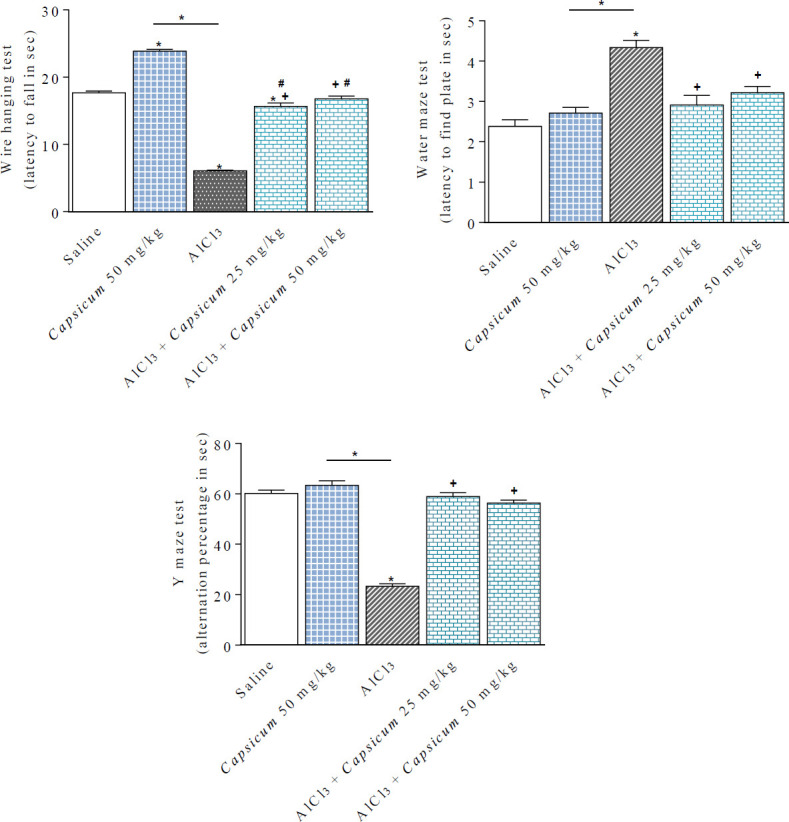
*Capsicum* alleviation of the impairment in muscle strength (wire hanging test) and memory performance (water maze and Y maze tests) in AlCl3 treated rats. *: *P*<0.05 vs saline control group and between different groups. +: *P*<0.05 vs AlCl_3_ treated group. #: *P*<0.05 vs *Capsicum* only (50 mg/kg)

**Figure 5:**
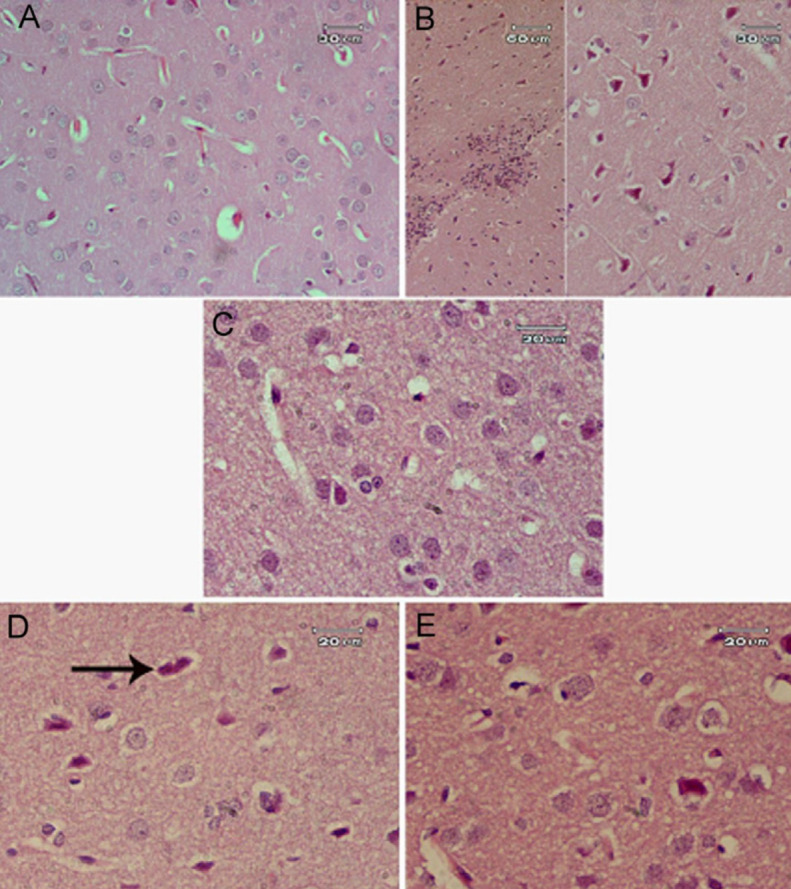
Representative photomicrographs of the rat cerebral cortex area after treatment with (A) Saline: showed normal structure. (B) AlCl_3_: showed aggregations of inflammatory cells (on the left side of the figure) and many small deeply stained neurons (on the right side). (C) *Capsicum* 50 mg/kg: showed normal neurons. (D) AlCl_3_ + *Capsicum* 25 mg/kg: some small deeply stained neurons (arrow) were still seen. (E) AlCl_3_ + *Capsicum* 50 mg/kg: showed only a few cells that appeared dark in color if compared with normal neurons

**Figure 6:**
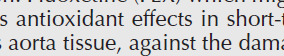
Representative photomicrographs of rat hippocampus after treatment with (A) Saline: showed normal structure of neurons in hippocampal area. (B) AlCl_3_: showed many small dark cells (arrow). (C) *Capsicum* 50 mg/kg: showed normally appearing neurons in structure and arrangement. (D) AlCl_3_ + *Capsicum* 25 mg/kg: showed normal thickened area but some neurons were still smaller in size and deeply stained (arrow). (E) AlCl_3 _+ *Capsicum* 50 mg/kg: showed that most of the neurons were normal but with few darkly stained cells (arrow)

**Figure 7:**
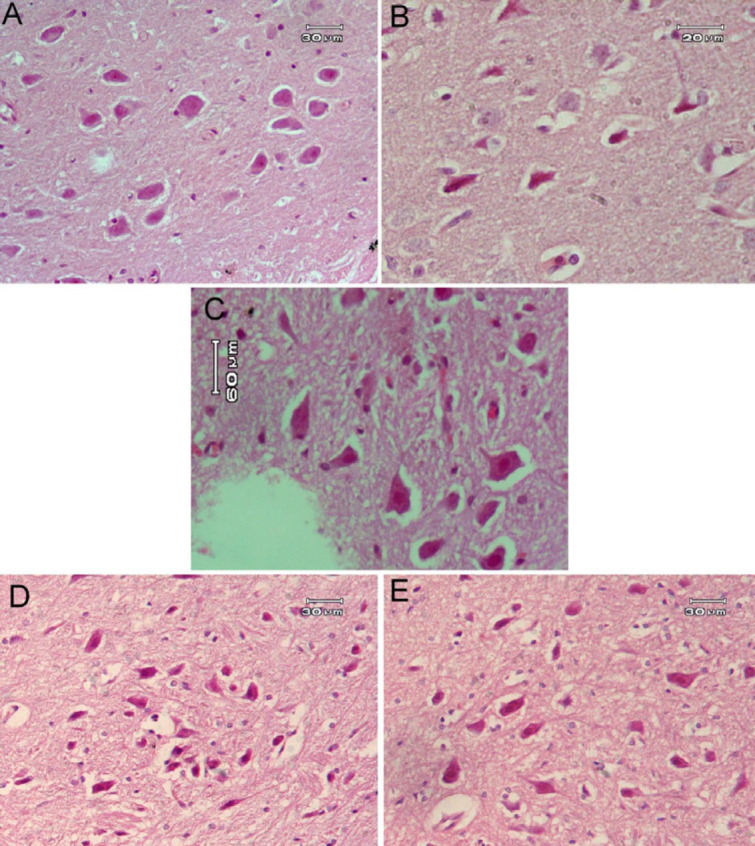
Representative photomicrographs of rat substantia nigra after treatment with: (A) Saline: showed the normal shape and size of the pigmented neurons. (B) AlCl3: showed size decrement of pigmented neurons. (C) *Capsicum* 50 mg/kg: showed a close to normal structure. (D) AlCl_3_ + *Capsicum* 25 mg/kg: showed a decrement in the pigmented neuron size, although their numbers were not significantly affected. (E) AlCl_3_ + *Capsicum* 50 mg/kg: showed normal-size neurons

## Results

***Lipid peroxidation***

It was found that 50 mg/kg *Capsicum* caused a 25.9% significant decrease in brain MDA compared with the control group (15.41 ± 0.39 vs 20.79 ± 0.85 nmol/g. tissue). Meanwhile, rats treated with AlCl_3_ alone for 2 months showed significantly increased brain MDA levels by 55.1% (32.25 ± 0.60 vs 20.79 ± 0.85 nmol/g. tissue) compared with their saline controls. In AlCl_3_/*Capsicum*-treated groups, MDA significantly decreased by 18.7%, and 26.9% (from 32.25 ± 0.60 to 26.23 ± 1.38 and 23.55 ± 0.91 nmol/g. tissue) (Figure 1).

***Nitric oxide***

Treatment with *Capsicum* 50 mg/kg didn’t alter the nitric oxide content in the brain. Brain nitric oxide was significantly elevated by 133.8% (42.72 ± 0.81 vs 18.27 ± 0.41 µmol/g. tissue) in AlCl_3_-treated rats compared with the control group. However, in AlCl_3_/*Capsicum*-treated rats, nitric oxide levels were decreased by 31.7% and 32.8% compared with the AlCl_3_ treated group (from 42.72 ± 0.81 in the AlCl*3* group to 29.36 ± 0.51 and 28.72 ± 0.65 µmol/g. tissue in the AlCl_3_/*Capsicum*-groups) (Figure 1).

***Reduced glutathione***

Compared with the saline control, brain GSH was significantly decreased by 43.7% in rats treated with AlCl_3_ (from 3.73 ± 0.18 to 2.1 ± 0.09 µmol/g. tissue in the AlCl_3_ group). 50 mg/kg *Capsicum* did not affect brain GSH levels. In contrast, rats treated with AlCl_3_/*Capsicum* exhibited significant increments in brain GSH content by 34.8% and 38.6%, respectively, compared with the AlCl_3_ treated group (2.83 ± 0.11 and 2.91 ± 0.13 *vs* AlCl_3_ value of 2.1 ± 0.09 µmol/ g. tissue) (Figure 1).

***Amyloid*** ***Aβ-peptide***

The concentration of amyloid Aβ-peptide was markedly and significantly increased by 259.2% in the brain of AlCl_3_-treated rats (10.49 ± 0.73 vs 2.92 ± 0.16 pg/ml). Administering 50 mg/kg *Capsicum* did not alter Aβ-peptide concentration. However, in rats treated with AlCl_3_ and *Capsicum* at (25 & 50 mg/kg), Aβ-peptide concentrations fell by 33.3% and 65.7%, respectively, in comparison with the AlCl_3_ treated group (7.0 ± 0.53 and 3.6 ± 0.27 *vs* 10.49 ± 0.73 pg/ml) (Figure 2).

***Interleukin-6***

Rats receiving AlCl_3_ injections exhibited a significant increment in brain IL-6 levels by 84.6% in comparison with their corresponding controls (14.29 ± 0.65 vs 7.74 ± 0.2 ng/ml). *Capsicum* (50 mg/kg) had no effect on IL-6. However, a significant reduction in IL-6 concentration by 21.9% and 59.5% was observed in AlCl_3_/*Capsicum*-treated groups, in comparison with the AlCl_3_ value (11.16 ± 0.29 and 9.93 ± 0.0.3 *vs*14.29 ± 0.65 ng/ml) (Figure 2). 

***Paraoxonase-1***

Brain PON-1 activity decreased by 36.0% in the AlCl_3_-treated group compared with the saline group (7.0 ± 0.15 *vs* 10.94 ± 0.27 kU/l). *Capsicum* (50 mg/kg) had no effect on PON-1 activity. However, in rats treated with AlCl_3_ and *Capsicum* (25 or 50 mg/kg), brain PON-1 activity was elevated by 21.7% and 46.6%, respectively, in comparison with the AlCl_3_ treated group (8.52 ± 0.26 and 10.26 ± 0.85 *vs* 7.0 ± 0.15 kU/l) (Figure 3).

***Acetylcholinesterase***

*Capsicum* (50 mg/kg) had no effect on AChE concentration in the brain (3.92 ± 0.26 vs 3.78 ± 0.06 ng/ml). Brain AChE concentration increased by 27.5% in the AlCl_3_ group in comparison with the saline group (4.82 ± 0.13 vs 3.78 ± 0.06 ng/ml). *Capsicum* had no significant effect on AChE in different groups treated with AlCl_3_ (Figure 3).

***Behavioral testing***

*Effect of capsicum in memory tests*

*Water maze test*

*Capsicum *at a dose of 50 mg/kg did not affect the time taken by rats to reach the hidden platform. On the other hand, AlCl_3_-treated rats exhibited significantly increased escape latency by 82.3% (4.34 ±0.18 vs 2.38 ± 0.16 sec) in comparison with the saline group. In contrast, rats given AlCl3/*Capsicum* at 25 or 50 mg/kg exhibited significant decrements in their escape latency times by 32.9% and 25.8% compared with AlCl_3_ treated group (2.91 ± 0.24 and 3.22 ± 0.15 vs 4.34 ± 0.18 sec) (Figure 4). 

*Y- maze test*

*Capsicum *given at 50 mg/kg had no effect on the percentage of spontaneous alternation. In rats receiving repeated injections of AlCl_3_, there was a significant decrease in the alternation percentage by 61.1% compared with their saline controls (23.4 ± 1.0 vs 60.1 ± 1.2 sec). In AlCl_3_/*Capsicum*-treated groups, the alternation percentage was restored to the saline control value (58.9 ± 1.5 and 56.4 ± 1.13 vs 60.1 ± 1.2 sec) (Figure 4). 

*Effect of capsicum on muscle strength*

Rats that received* Capsicum *at 50 mg/kg spent more time in the wire-hanging test. Rats treated with AlCl_3_ exhibited a significant decrease in the latency to fall by 65.5% compared with their saline controls (6.1 ± 0.1 vs 17.69 ± 0.24 sec). In AlCl_3_-treated rats, significant increments in the latency fall by 156.2% and 175.1% due to the use of *Capsicum *at 25 or 50 mg/kg compared with the AlCl_3_ control value (15.63 ± 0.54 and 16.78 ± 0.43 vs 6.1 ± 0.1 sec) (Figure 4). 

*Histopathological results*

It was clear that AlCl_3 _had a damaging effect on neurons in several brain regions (cerebral cortex, hippocampus, and substantia nigra). Many small darkly-stained neurons appeared in examined sections (Figures 5B, 6B, and 7B) in comparison with the normal tissue structure of these areas (Figures 5A, 6A, and 7A). The administration of *Capsicum* at 50 mg/kg had no effects in these brain areas (Figures 5C, 6C, and 7C). *Capsicum (*25 or 50 mg/kg) given to AlCl_3_-treated rats had an ameliorating effect on brain tissue damage but with no marked difference between the two doses used in this study (Figures 5D-E, 6D-E, and 7D-E).

## Discussion

The treatment of rats with AlCl_3_ in this study caused increment of brain oxidative stress due to elevation of nitric oxide and lipid peroxidation along with depletion of the anti-oxidant reduced glutathione. In addition, there were significant increases in brain AChE, IL-6, and Aβ-peptide levels. Treatment with AlCl_3_ impaired memory performance and neuromuscular strength. The histological study indicated that AlCl_3_ caused neuronal degeneration in a number of brain regions. The administration of hot pepper extract one month after starting AlCl_3_ resulted in marked alleviation of the biochemical, behavioral, and neuronal damage in the brain of AlCl_3_-treated rats.

In this study treatment with AlCl_3_ showed induced oxidative stress in rat brains as evidenced by the increment of malondialdehyde and depletion of the anti-oxidant and radical scavenger reduced glutathione (35). These observations are in agreement with other published studies that found increased brain lipid peroxidation and decreased reduced glutathione and reduced activities of the antixidants enzymes catalase and superoxide dismutase in the brain of AlCl_3_-treated rats and mice (36, 37). Oxidative damage was considered an important mechanism that underlies neurotoxicity caused by Al, by displacing iron from its binding sites, thereby, increasing the availability of this transition metal to participate in cell-damaging redox reactions (38, 39). In this study, the use of hot pepper extract was found to alleviate the increase in brain malondialdehyde and to elevate reduced glutathione in the brain of AlCl_3_ rats which suggests that an anti-oxidant process collaborated in the neuroprotection by the extract.

We also found a marked increase in brain nitric oxide content in the brain of AlCl_3_-treated rats which is supported by other studies (36, 40). Neurotoxic effects of high concentrations of nitric oxide are caused by peroxynitrite (ONOO-) and reactive nitrogen oxides, capable of oxidation, nitration, and nitrosylation reactions (41, 42). Meanwhile, inhibiting nitric oxide synthases was reported to provide neuroprotection in the brain of AlCl_3_-treated animals, thereby suggesting an important action of nitric oxide in Al-induced neurotoxicity (43). In this study, we found that the administration of hot pepper extract in AlCl_3_-treated animals caused a significant decrease in brain nitric oxide, thereby suggesting that inhibition of nitric oxide may be involved in the neuroprotective effect of hot peppers.

 We also noticed a significant and marked increment in brain proinflammatory cytokine IL-6 level which suggests the presence of brain inflammatory response, a mechanism that contributes to neuronal damage. In addition, we showed a significant elevation in AChE concentration in the brain of AlCl_3_-treated rats which is in agreement with previous studies (40, 44). Other researchers reported a decrement in choline acetyltransferase in the tissue homogenate of the hippocampus and forebrain as well as increased AChE activity in caudate after intravenous injection of AlCl_3 _(45). The presence of extracellular Aβ deposits was considered the most important neuropathologic hallmark of AD and was held responsible for the initiation of a cascade of events that culminate in the death of neurons and disease manifestations (4). In this study, using ELISA, we found a significant and marked increase in Aβ concentrations in the brain of rats that received AlCl_3_ injections which is in accordance with previously published observations (46). Here, we report that treatment with hot pepper extract was associated with marked decrement in the elevated Aβ peptide concentrations in the brain of AlCl_3_-treated rats. We suggest that the hot peppers interfere with the pathogenetic pathway leading to the increased production and/or accumulation of Aβ peptides in brain tissue. 

Paraoxonase-1 (PON-1) is an esterase and lactonase which has the ability to hydrolyze some of the organophosphorus insecticides, nerve agents, lipid hydroperoxides as well as other xenobiotics (47). The enzyme is considered to play a neuronal protective role by virtue of its anti-oxidant and anti-inflammatory properties (48). In this study, PON-1 activity was significantly decreased in rat brains after AlCl_3 _treatment, which is in agreement with previous studies (40). Paraoxonase-1 is inactivated by oxidative stress via interaction between the enzyme-free sulfhydryl group and oxidized lipids (49), while anti-oxidants e.g., vitamin C and E increase enzyme activity (50) which provides a plausible explanation for our present findings. Lower levels of oxidative stress due to using hot peppers may be the cause of the increased activity of brain PON-1 in AlCl_3_-treated rats, preventing activation of the enzyme. 

Our experimental model was designed to investigate the potential for hot peppers to interfere with the ongoing neurodegeneration caused by AlCl_3_ and the underlying pathogenetic mechanisms involved. Thus, hot pepper extract was given to rats one month after the establishment of neuronal damage and the administration of AlCl_3_ continued so as to maintain the neurodegenerative process. Under these circumstances, treatment with hot pepper was shown to decrease neuroinflammation (increased IL-6) and oxidative stress in the brain, prevent the impairment in muscle strength and memory processing, and offered neuronal protection. Other researchers showed, in diabetic rats that received β-amyloid infusion into the hippocampus, that supplementation of diet with ethanolic extracts of red peppers of moderate or high pungency, prevented memory deficit. The extracts which were given at doses equivalent to 3 g/day in humans were found to inhibit β-amyloid plaque accumulation and tau phosphorylation (51). The neuroprotective effects described in this study for hot pepper are also supported by previous observations in which hot pepper protected brain neurons from spongiform degeneration, neuronal apoptosis, and necrosis that occurred in insulin-induced hypoglycemia (21). Hot pepper also protected pigmented substantia nigra cells, and cortical and hippocampus neurons from the toxic effects of rotenone (20). In these studies, hot pepper showed anti-oxidant activity as indicated by the decrement of nitric oxide level and lipid peroxidation in the brain, and restoration of GSH content. It also exhibited anti-inflammatory action by decreasing brain 5-lipoxygenase. 

Capsaicin is the major constituent that accounts for the pungency of hot red or green peppers of the plant genus *Capsicum* (16, 17). These popular food ingredients were shown to benefit cognitive functioning in man and experimental animals. In their study, Liu *et al*. (22) found a positive correlation between capsaicin content in diet and cognitive function in subjects who were 40 years of age or older. Capsaicin in the diet was also found to correlate negatively with Aβ_40_ and total Aβ serum levels (though not with Aβ_42_). Wang *et al*. (23) used the APP/PSI genetic mouse model of AD and reported prevention of cognitive decline and Aβ deposits in the brain by feeding with chow supplemented with 0.01% capsaicin (from 3 to 9 months of age). The authors estimated the daily capsaicin intake at ∼ 30 mg/kg for mice in the study. β-Secretase and γ-secretase cause sequential breakdown of the amyloid precursor protein (APP) to produce Amyloid-β peptides (Aβ) with Aβ-42 being the species most involved in AD pathogenesis. A third α-secretase pathway that cleaves APP in the middle of the Aβ domain precludes the formation of Aβ (52). Capsaicin may prevent Aβ deposits by increasing the non-amyloidogenic processing of APP via α-secretase (23). 

Capsaicin acts on the transient receptor potential vanilloid type 1 cation channel (TRPV1) (18). These channel receptors are expressed on capsaicin-sensitive sensory nerves, dorsal root ganglia, and several brain regions on neurons, astrocytes, and pericytes (53). TRPV1 channels act as a polymodal detector of nociceptive information that responds to noxious stimuli such as capsaicin, heat (> 43 °C), protons, bradykinins, lipoxygenase products of arachidonic acid, the endocannabinoids anandamide, and *N*-arachidonoyl-dopamine (19, 54). In the brain, TRPV1 has a modulatory role in neurotransmitter release, synaptic plasticity, and neuroinflammation (55). Whether the effects of hot peppers in the diet on cognition and Aβ deposition are mediated by its pungent principle capsaicin and hence TRPV1 stimulation cannot be ruled out (23). Based on a capsaicin content of 1.2% in the present sample of hot peppers, the daily intake of capsaicin in countries with the highest consumption of hot peppers has been estimated to be 0.27-0.81 g/kg (56). Capsaicin has been shown to reach the brain at ng/g concentrations after intravenous injection of 2 mg/kg in rats (57). These concentrations of capsaicin were shown to stimulate TRPV1 receptors (19). It is worth mentioning here that capsaicin given systemically at small doses ∼ 0.15–1.5 mg/kg, was seen to have neuroprotective action in several experimental models of neuronal damage like global cerebral ischemia in the brain(58), Parkinson’s disease (59), and endotoxemia (60, 61). In these studies, capsaicin alleviated brain oxidative stress, serum nitric oxide (61), plasma nitric oxide, IL-6, and tumor necrosis factor-alpha (TNF-α) (60). Capsaicin thus may inhibit amyloidogenesis by altering the oxidative and inflammatory milieu that favors Aβ deposition. 

Hot peppers are also rich in anti-oxidants like carotenoids which are responsible for the color of these fruits. Capsanthin, capsorubin, and cryptocapsin are responsible for the red color while β-carotene, zeaxanthin, violaxanthin, and β-cryptoxanthin confer the yellow color. *Capsicum* may contain up to 3.2 g carotenoids/100 g dry weight (62) besides other anti-oxidants like vitamin C (76.4 mg/100 g), vitamin A (41.6 mg/100 g), α-tocopherol or vitamin E (29.8 mg/100 g) (63), phenolic compounds, and flavonoids (64). Studies found that compared with cognitively intact individuals, α-tocopherol and the carotenoids β-carotene, β-cryptoxanthin, lutein, lycopene, retinol, and zeaxanthin were significantly lower in sera from patients with AD (65). This may suggest a role for deficiency in these nutrients in pathogenetic mechanisms leading to cognitive decline in AD. It was also shown that carotenoids and vitamin constituents of *Capsicum* may benefit cognition and memory functioning in the aged. Thus, subjects with high vitamin E intake showed a 36% reduction in the rate of decline of their cognitive scores when compared with those with the lowest intake (66). Power *et al*. (67) found that compared with a placebo, supplementation of diet with lutein, zeaxanthin, and meso-zeaxanthin for 12 months significantly improved memory in healthy subjects. Moreover, a higher intake of total carotenoids (median intake of 24.8 mg/day), in particular lutein/zeaxanthin, was found to result in a 48% lower risk for developing AD and less neurofibrillary tangles and amyloid plaques (68). 

## Conclusion

The present study demonstrated that hot pepper extract reduced AlCl_3_-induced oxidative stress and increased IL-6 and Aβ concentrations in the brain. Hot pepper improved memory impairment and neuromuscular strength and prevented neuronal damage. These findings indicate that hot peppers, a popular component of human food can be of value in maintaining cognitive function and in preventing neurodegeneration in the AD brain.

## Authors’ Contributions

OMEAS Conceived the study, designed, and performed data analysis; MESES Prepared plant material, and performed experiments and data analysis; ERY Helped with biochemical analysis; NS did histopathological studies and interpretation of results; OMEAS, MESES, ERY, and NS Prepared the manuscript, and revised and approved the final version to be published.

## Ethical Approval

Animal procedures followed the guidelines of the of the National Research Centre for the Use of Animals in Experimental Studies and the Guide for Care and Use of Laboratory Animals by the U.S. National Institutes of Health (Publication No. 85-23, revised 1996). 

## Financial Support

This work was not supported by research grants.

## Conflicts of Interest

The authors declare no conflicts of interest.
